# Acute Tachycardia Increases Aortic Distensibility, but Reduces Total Arterial Compliance Up to a Moderate Heart Rate

**DOI:** 10.3389/fphys.2018.01634

**Published:** 2018-11-19

**Authors:** Yunlong Huo, Huan Chen, Ghassan S. Kassab

**Affiliations:** ^1^PKU-HKUST Shenzhen-Hongkong Institution, Shenzhen, China; ^2^Department of Mechanics and Engineering Science, College of Engineering, Peking University, Beijing, China; ^3^California Medical Innovations Institute, San Diego, CA, United States

**Keywords:** acute tachycardia, total arterial compliance, arterial distensibility, pulse wave velocity, Windkessel model, Womersley model

## Abstract

**Background:** The differential effects of rapid cardiac pacing on small and large vessels have not been well-established. The objective of this study was to investigate the effect of pacing-induced acute tachycardia on hemodynamics and arterial stiffness.

**Methods:** The pressure and flow waves in ascending aorta and femoral artery of six domestic swine were recorded simultaneously at baseline and heart rates (HR) of 135 and 155 beats per minutes (bpm) and analyzed by the models of Windkessel and Womersley types. Accordingly, the flow waves were simultaneously measured at carotid and femoral arteries to quantify aortic pulse wave velocity (PWV). The arterial distensibility was identified in small branches of coronary, carotid and femoral arteries with diameters of 300–600 μm by *ex vivo* experiments.

**Results:** The rapid pacing in HR up to 135 bpm reduced the total arterial compliance, stroke volume, systemic pulse pressure, and central systolic pressure by 36 ± 17, 38 ± 26, 29 ± 16, and 23 ± 12%, respectively, despite no statistical difference of mean aortic pressure, cardiac output, peripheral resistance, and vascular flow patterns. The pacing also resulted in a decrease of distensibility of small muscular arteries, but an increase of aortic distensibility. Pacing from 135 to 155 bpm had negligible effects on systemic and local hemodynamics and arterial stiffness.

**Conclusions:** There is an acute mismatch in the response of aorta and small arteries to pacing from basal HR to 135 bpm, which may have important pathological implications under chronic tachycardia conditions.

## Introduction

Epidemiologic data show that hypertension and atrial fibrillation (AF) often coexist (Dzeshka et al., 2017; Andreadis and Geladari, 2018; Verdecchia et al., 2018). Patients with high blood pressure (BP) show higher risk of developing AF by 50% in men and 40% in women (Benjamin et al., 1994) while >60% of patients with AF have hypertension (Verdecchia et al., 2018). Although multiple clinical studies have shown a direct relationship between BP levels and the risk of AF, the form of the relationship remains unknown (Conen et al., 2009; Grundvold et al., 2012; Verdecchia et al., 2012a,b). Patients with high BP, however, do not benefit from the pharmacological heart rate (HR) lowering (Rimoldi et al., 2016). It is also unclear whether the pharmacological BP control can relieve the incidence and progression of AF (Manolis et al., 2012; Verdecchia et al., 2016; Whelton et al., 2018). Hence, one objective of this study is to quantify the relationship between acute tachycardia and systemic hemodynamics in normotensive swine.

A large number of epidemiological studies have shown that elevated HR increases cardiovascular morbidity and mortality such that high HR is considered as a prognostic factor for the cardiovascular disease independent of other risk factors (e.g., hypertension, hyperlipidemia, diabetes, and so on) (Bergel, 1961; Palatini and Julius, 2004; Diaz et al., 2005; Fox et al., 2007; Lonn et al., 2010; Fox and Ferrari, 2011). The pathophysiology of elevated HR for cardiovascular disease is potentially involved in the decrease of arterial distensibility because a long-term elevated arterial stiffness can be an important determinant of the development and progression of hypertension (Stefanadis et al., 1998; O'Rourke and Hashimoto, 2007; Fox and Ferrari, 2011; Safar, 2018). There is also debate on the mechanical response of large conduit arteries to acute tachycardia (Stefanadis et al., 1998; Liang et al., 1999; Wilkinson et al., 2000; Albaladejo et al., 2001; Lantelme et al., 2002; Haesler et al., 2004) and lack of studies on the mechanical response of small arteries to heart rate changes. Based on the *in vivo* and *ex vivo* measurements, the second objective of this study is to investigate the effects of rapid pacing on arterial distensibility in aorta and small arteries of normotensive swine, which can enhance the understanding of arterial stiffness relevant to elevated HR.

Total arterial compliance and peripheral resistance are two key parameters that have been widely used to characterize systemic hemodynamics (Liu et al., 1986; Stergiopulos et al., 1994, 1995; Westerhof et al., 2009; Nichols and McDonald, 2011). Based on the classical two-element Windkessel model, pulse pressure method has been used to estimate the total arterial compliance accurately (Stergiopulos et al., 1994). To generalize the model, researchers have added more elements (e.g., three-element Windkessel model) (Westerhof et al., 1971; Latson et al., 1988; Laskey et al., 1990) and incorporated non-linearities (Burattini et al., 1987; Li et al., 1990). Moreover, the three-element Windkessel model can be used to quantify aortic characteristic impedance, which is proportional to arterial stiffness (Huo and Kassab, 2006, 2007). On the other hand, pulse wave reflections derived from transmission theory (Westerhof et al., 1972, 2006; Westerhof and Westerhof, 2013) are sensitive to heart rate and peripheral vasculature (Quick et al., 1998). The analytic model of Womersley type features transient flow patterns in aorta and peripheral arteries (Zheng et al., 2010). The third objective of this study is to use the models of Windkessel and Womersley types to perform systemic and local hemodynamic analyses.

We hypothesize that pacing-induced acute tachycardia decreases the acute stiffness of aorta and increases the acute stiffness of small arteries in some pressure range, which results in an acute mismatch. Moreover, pacing-induced acute tachycardia reduces the total arterial compliance, systemic pulse pressure (PP), central systolic pressure, and stroke volume (SV) in some pressure range despite no statistical difference of mean aortic pressure (MAP), cardiac output (CO), peripheral resistance, and transient flow patterns in aorta and peripheral arteries. To test these hypotheses, the simultaneous measurement of blood pressure, flow, and cross-sectional area (CSA) was performed in ascending aorta and femoral artery of six domestic swine at baseline and during right atrial pacing to HR of 135, 155, and 170 beats per minutes (bpm). Analytic models of Windkessel and Womersley types were used to carry out transient hemodynamic analysis based on these experimental measurements. Aortic pulse wave velocity (PWV) was determined using two simultaneously measured flow waves. In addition to the *in vivo* experiments, the *ex vivo* pulsatile pressure-diameter measurements were demonstrated in small branches of coronary, carotid and femoral arteries when the frequency was varied in the range of 1–3 Hz (mimicking the HR of 60–180 bpm). The physiological implications of initial rapid pacing are discussed along with the limitations and significance of the study.

## Materials and methods

### Animal preparation

Studies were performed on six domestic swine weighing 71 ± 8 kg for *in vivo* hemodynamic pacing measurements and six controls with similar weights for *ex vivo* measurements of dynamic pressure and diameter waves in small arteries of 300–600 μm in diameter. All animal experiments were performed in accordance with Indiana University, Purdue University, Indianapolis, consistent with the NIH guidelines (Guide for the care and use of laboratory animals) on the protection of animals used for scientific purposes. The experimental protocols were approved by the Institutional Animal Care and Use Committee of Indiana University.

The animal preparation was similar to previous studies (Zheng et al., 2010; Huo and Kassab, 2015). Briefly, surgical anesthesia was induced with TKX (Telaxol 500 mg, Ketamine 250 mg, Xylazine 250 mg) and maintained with 2% isoflurane. The animal was intubated and ventilated with room air and oxygen by a respiratory pump. A side branch from the left jugular vein was dissected and cannulated with 7Fr. sheath for administration of drugs (e.g., heparin, lidocaine, levophed, papaverine, and saline). The right femoral artery was cannulated with a 7Fr. sheath and connected to a pressure transducer (Summit Disposable Pressure Transducer, Baxter Healthcare; error of ±2% at full scale) for monitoring arterial blood pressure.

The right jugular vein was exposed and cannulated with a 9Fr. sheath for the advancement of the pacing lead into the right atrium. The pacing lead (Medtronic 5568) was screwed into the wall of right atrium and connected to a pacemaker (Medtronic Enpulse E2DR01) which was placed into a subcutaneous pocket. The ascending and descending aortas and femoral artery were dissected. Perivascular flow probes (Transonic Systems Inc.; relative error of ±2% at full scale) were mounted on these arteries to measure the volumetric flow rate. Flow and pressure were continuously recorded using a Biopac MP 150 data acquisition system. The cross-sectional area (CSA) of arteries was determined using ultrasound (Philips IE33 ultrasound system).

### *In vivo* measurements

The heart rate was paced to 135, 155, and 170 bpm as compared with basal HR of about 90 bpm (range of 80–105 bpm). The animals were allowed to recover to basal HR between consecutive pacing sessions. Electrocardiography (ECG) signals were used to monitor HR. The aortic, carotid and femoral flow rates were measured simultaneously. The femoral arterial pressure was measured by the pressure transducer connected to the 7Fr. sheath. The aortic pressure was determined when the sheath was advanced to aorta under fluoroscopy.

We have shown an abrupt decrease of blood pressure and flow after rapid pacing (initial period) and then recovered close to baseline after about 3–5 min of pacing (recovery period) (Zheng et al., 2010). Since the present study only considered the hemodynamic analysis in the recovery period, the flow and pressure waves as well as ECG signals were continuously recorded for about 10 min under each HR by a data acquisition system (MP 150, Biopac Systems Inc.). Finally, animals were euthanized by an injection of pentobarbital sodium (300 mg/kg).

### *Ex vivo* measurements in small arteries

Similar to previous studies (Huo et al., 2012, 2013; Lu et al., 2017), arteries with diameters of 300–600 μm were dissected free of periarterial tissues. Two black marks were made at the two ends of the artery with waterproof India ink. The image of the vessel was displayed on screen with a CCD camera mounted on the dissection microscope to determine the *in vivo* length (i.e., the length between the two black marks) and then isolated from various positions (i.e., small branches of coronary, carotid, and femoral arteries) and dissected free of fat and connective tissue after euthanasia. The side branches were ligated with suture under dissection microscope in 4°C HEPES PSS (physiological saline solution). An artery specimen was mounted on the two cannulas in an organ bath chamber containing PSS solution. One cannula was connected with a pressure transducer through a Y tube, while the other was connected with a bottle of PSS solution that induced sawtooth flow wave by a piston pump. The temperature in the bath and bottle was gradually increased to 37°C in 10 min. The vessel was stretched close to the *in vivo* length (axial stretch ratio approximately equals to 1.4). The image of the vessel was displayed on screen with a CCD camera mounted on a stereo microscope and the changes of outer diameter were measured with dimensional analysis software (DIAMTRAK 3+, Australia). The pressure transducer and diameter tracings were interfaced into a computer by a data acquisition system (MP 150, Biopac Systems Inc.), which monitored transient changes of pressure and diameter. The time-averaged pressure over a cardiac cycle equaled to 80 mmHg. We determined the changes of the arterial distensibility as the frequency increases from 1 to 3 Hz (by a step of 0.5 Hz) to mimic HR from 60 to 180 bpm.

### Windkessel analysis

Based on the *in vivo* measurements of pressure and flow waves in aorta and femoral artery, we determined the time-averaged pressure and flow over a cardiac cycle (P_mean_ and Q_mean_). MAP and PP equal to P_mean_ and the difference of systolic and diastolic pressures, respectively, in ascending aorta. The CO was computed by Q_mean_×60 s and the SV was calculated as the ratio of CO to HR. The classical two-element Windkessel model (Stergiopulos et al., 1994) is written as:

(1) $$\begin{array}{r} Z\left(\omega \right)=\frac{R}{1+j\omega RC} \end{array}$$

where *Z*(ω) is the input impedance, ω the angular frequency after Fourier transformation, C the total arterial compliance [ = dV(t)/dP(t), where V(t) is the blood volume of the arterial system and P(t) is the blood pressure of the most proximal artery], and R the peripheral resistance (= P_mean_/Q_mean_). The peripheral resistance was obtained directly from the measured pressure and flow waves and the total arterial compliance was estimated by the pulse pressure method (Stergiopulos et al., 1994). The aortic characteristic impedance (Z_c_) was determined using the three-element Windkessel model (Westerhof et al., 1971) as:

(2) $$\begin{array}{r} Z\left(\omega \right)={\text{Z}}_{\text{c}}+\frac{\text{R}-{\text{Z}}_{\text{c}}}{1+\text{j}\omega \left(\text{R}-{\text{Z}}_{\text{c}}\right)\text{C}} \end{array}$$

where *Z*_*c*_ is the characteristic impedance, i.e., an important parameter to address the relationship between pulsatile pressure and pulsatile flow in an artery when pressure and flow waves are not influenced by wave reflection. Since R and C are determined by experimental measurements and pulse pressure method of Equation (1), *Z*_*c*_ is estimated by minimizing the error between the input impedance by Fourier transformation of the ratio of the measured pressure to flow waves $\left[\frac{p_{measured}\left(t\right)}{q_{measured}\left(t\right)}\right]$ and the one predicted by Equation (2), i.e., minimizing $\left[\frac{\left|Z_{measured}\left(\omega \right)-Z_{estimated}\left(\omega \right)\right|}{\left|Z_{measured}\left(\omega \right)\right|}\right]$.

### Forward and backward waves

The forward [*p*_*forward*_(*t*), *q*_*forward*_(*t*)] and backward [*p*_*backward*_(*t*), *q*_*backward*_(*t*)] pressure and flow waves are given as Westerhof et al. (1972):

(3) $$\begin{array}{r} {\text{p}}_{\text{forward}}\left(\text{t}\right)=\left[{\text{p}}_{\text{measured}}\left(\text{t}\right)+{\text{Z}}_{\text{c}}\cdot {\text{q}}_{\text{measured}}\left(\text{t}\right)\right]/2;\\ {\text{q}}_{\text{forward}}\left(\text{t}\right)={\text{p}}_{\text{forward}}\left(\text{t}\right)/{\text{Z}}_{\text{c}} \end{array}$$

(4) $$\begin{array}{r} {\text{p}}_{\text{backward}}\left(\text{t}\right)=\left[{\text{p}}_{\text{measured}}\left(\text{t}\right)-{\text{Z}}_{\text{c}}\cdot {\text{q}}_{\text{measured}}\left(\text{t}\right)\right]/2;\\ {\text{q}}_{\text{backward}}\left(\text{t}\right)=-{\text{p}}_{\text{backward}}\left(\text{t}\right)/{\text{Z}}_{\text{c}} \end{array}$$

The forward pressure and flow waves have the same shape. The backward pressure and flow waves also have the same shape, but are inverted with respect to another.

### Womersley analysis

Similar to a previous study (Zheng et al., 2010), the equation for the pulsatile flow velocity profile across the lumen, u(*r, t*), is given as:

(5) $$\begin{array}{r} \text{u(}r,t\text{)}=\text{REAL}\left(\frac{2Q\left(0\right)\left(R^{2}-r^{2}\right)}{\pi R^{4}}+\overset{\infty }{\underset{\omega =1}{\sum }}\frac{\frac{Q\left(\omega \right)}{\pi R^{2}}\cdot \left(1-\frac{J_{0}\left(\Lambda r/R\right)}{J_{0}\left(\Lambda \right)}\right)}{1-\frac{2J_{1}\left(\Lambda \right)}{\Lambda J_{0}\left(\Lambda \right)}}e^{i\omega t}\right) \end{array}$$

where *r* is the radial coordinate, *R* is the radius of artery, Λ^2^ = *i*^3^α^2^, ${\text{q}}_{\text{measured}}\text{(t)}=Q\left(\omega \right)e^{i\omega t}$, *J*_0_is a Bessel function of zero order and first kind, and *J*_1_ is a Bessel function of first order and first kind. Accordingly, wall shear stress (WSS), τ(*R, t*), and oscillatory shear index (OSI) for pulsatile blood flow can be written as:

(6) $$\begin{array}{r} \tau \left(R,t\right)=\text{REAL}\left(\frac{4\mu }{\pi R^{3}}Q\left(0\right)-\overset{\infty }{\underset{\omega =1}{\sum }}\frac{\frac{\mu Q\left(\omega \right)}{\pi R^{3}}\cdot \frac{\Lambda J_{1}\left(\Lambda \right)}{J_{0}\left(\Lambda \right)}}{1-\frac{2J_{1}\left(\Lambda \right)}{\Lambda J_{0}\left(\Lambda \right)}}e^{i\omega t}\right) \end{array}$$

(7) $$\text{OSI}=\frac{1}{2}\left(1-\frac{\left|\frac{1}{T}\int_{0}^{T}\tau \left(R,t\right)\right|}{\frac{1}{T}\int_{0}^{T}\left|\tau \left(R,t\right)\right|}\right)$$

The viscosity (μ) and density (ρ) were assumed to be 4.0 cp and 1.06 g/cm^3^, respectively. The forward and backward WSS were computed, based on *q*_*forward*_(*t*) and *q*_*backward*_(*t*), respectively.

### PWV and dynamic elastic modulus

Aortic PWV is the velocity at which the arterial pulse propagates through the vessel and used clinically as a measure of arterial stiffness. Similar to a previous study (Mohiaddin et al., 1993), we determined aortic PWV (PWV = L/ΔT, where L is the distance between carotid and femoral arteries and ΔT is the pulse transit time). The distance, L, was calculated as the distance between the sternal notch and the carotid site subtracted from the distance between the sternal notch and the femoral site using the subtraction method (Butlin et al., 2013) while the pulse transit time, ΔT, was computed using the foot-to-foot method based on the simultaneously measured carotid and femoral flow waves (Mohiaddin et al., 1993).

When tissue is modeled by Kelvin-Voigt model (Bergel, 1961), the vessel dynamic elastic modulus can be written as:

(8) Edyn=E'+jηω=Dmean| Δp ΔD|cosϕ+jηω

where $\left|\frac{\;\Delta \text{p}}{\;\Delta \text{D}}\right|$ is the amplitude ratio of sinusoidal pressure and diameter waves and $\phi =\tan^{-1}\left(\frac{\eta \omega }{{\text{E}}^{\;}}\right)$ is the phase lag of outer diameter behind pressure. Since ϕ is small (< 0.2 rad), ${\text{E}}_{\text{dyn}}≅\text{E}$ such that the dynamic elastic modulus ($≅{\text{D}}_{\text{mean}}|\frac{\;\Delta \text{p}}{\;\Delta \text{D}}|$) can be determined by Fourier transformation of the *in vitro* measured pulsatile waves. Furthermore, the arterial distensibility ($=\frac{2}{dynamic\;elastic\;modulus}$) was computed in small branches of coronary, carotid and femoral arteries.

### Data analysis

The measurements were repeated five times and averaged at each HR per animal. The mean and standard deviation (mean ± SD) were computed by averaging over all animals in each HR group. One Way Repeated Measures ANOVA (SigmaStat 3.5) was used to compare the various parameters (e.g., R, C, CO, SV, PWV, Z_c_, CSA, etc.) between baseline and various HR, where p- < 0.05 represented statistically significant differences.

## Results

**Figure 1:**
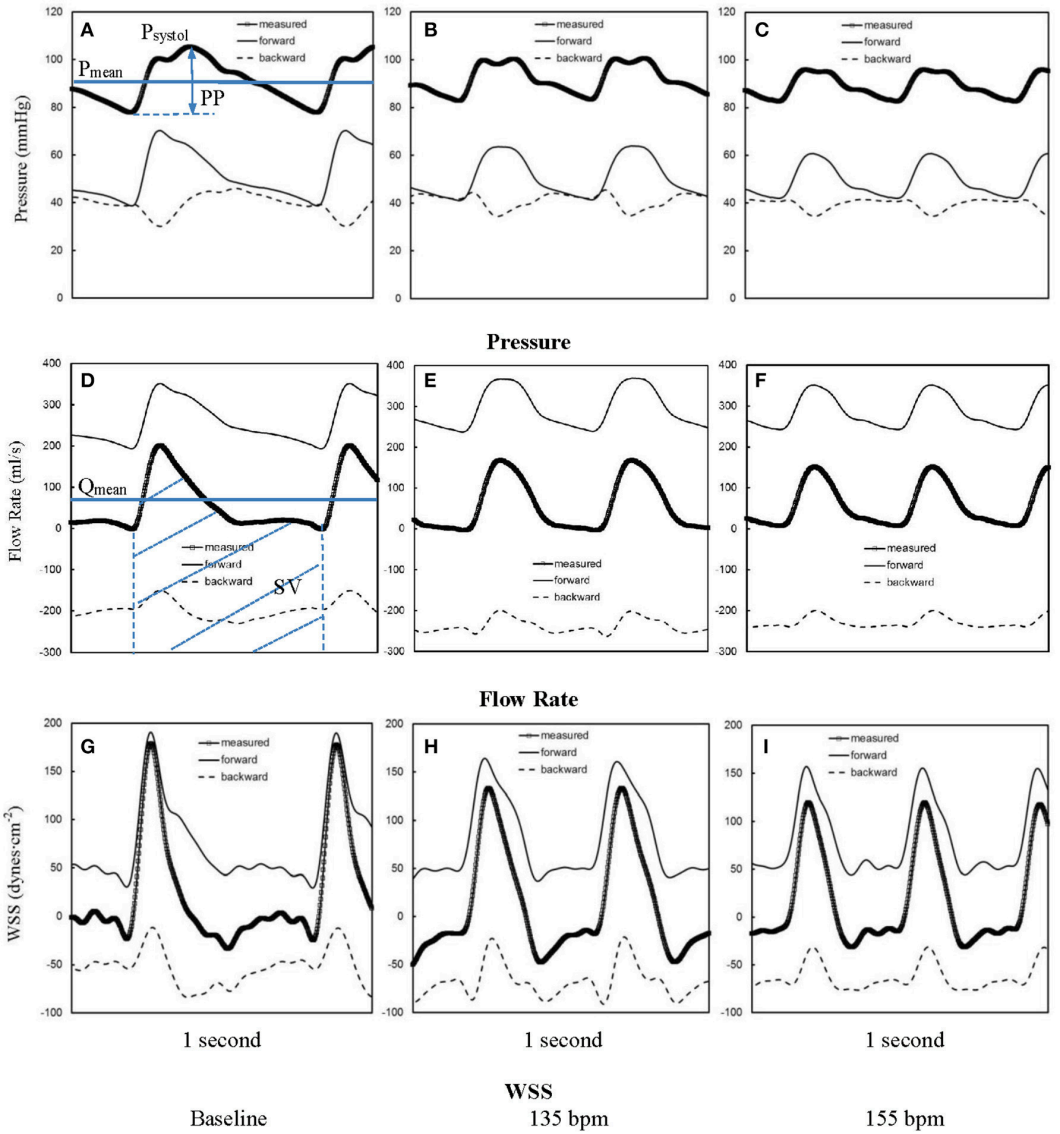
Pressure waves in ascending aorta **(A)** at baseline and after the heart rate was paced to **(B)** 135 bpm and **(C)** 155 bpm. **(D–F,G–I)** refer to flow and WSS waves, respectively, in ascending aorta corresponding to **A–C**. The marked, solid, and dash lines represent the measured, forward, and backward waves, respectively.

**Figure 2:**
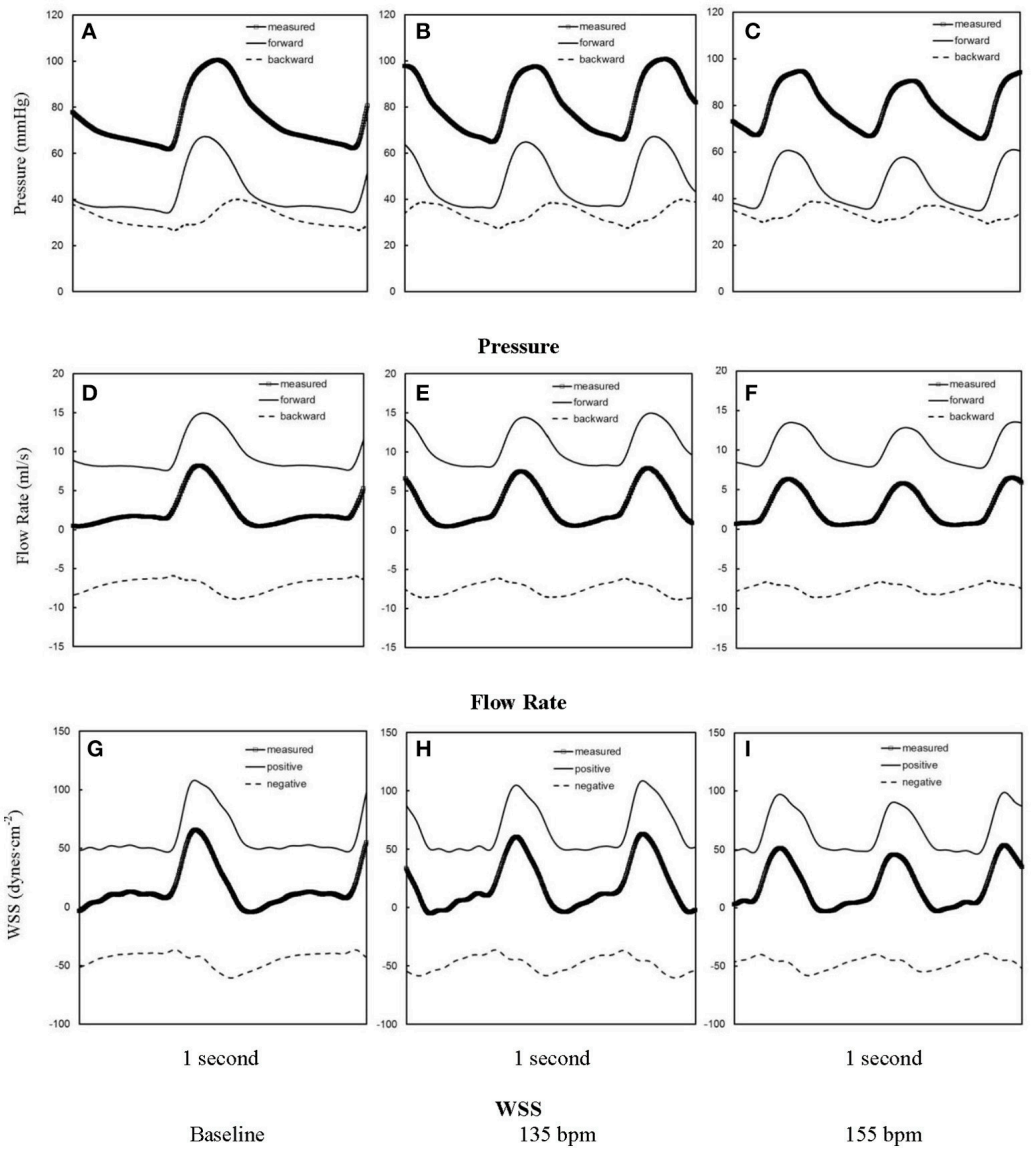
Pressure waves in femoral artery **(A)** at baseline and after the heart rate was paced to **(B)** 135 bpm and **(C)** 155 bpm. **(D–F,G–I)** refer to flow and WSS waves, respectively, in femoral artery corresponding to **(A–C)**. The marked, solid, and dash lines represent the measured, forward, and backward waves, respectively.

Figures 1A–C show pressure waves in ascending aorta at baseline and after HR was paced to 135 bpm and 155 bpm, where the marked, solid, and dash lines represent the measured, forward, and backward pressure waves, respectively. Accordingly, Figures 1D–F show flow waves and Figures 1G–I show WSS waves in ascending aorta at different HR. Figures 2A–I show pressure, flow, and WSS waves in femoral artery at baseline, 135 bpm, and 155 bpm in correspondence with Figures 1A–I. The ascending aorta and femoral artery of swine had MAP of 91 ± 6 and 86 ± 7 mmHg (averaged over 6 animals), respectively, in the supine position regardless of HR. The CO was 5.9 ± 3.6 and 5.6 ± 3.3 L/min (averaged over 6 animals) at baseline and 135 bpm, respectively. As shown in Figures 1A,D, SV, systemic PP, and central systolic pressure were reduced by 38 ± 26%, 29 ± 16%, and 23 ± 12% (averaged over 6 animals, *p* < 0.05), respectively, as HR increased from baseline to 135 bpm. The peripheral resistance was relatively unchanged (*p* > 0.25) as HR increased from baseline to 135 bpm while the total arterial compliance distal to aorta and femoral artery was decreased by 36 ± 17 and 29 ± 14% (*p* < 0.05), respectively, as shown in Table 1. There was no statistical difference in the total arterial compliance, systemic PP, central systolic pressure, CO, and SV between 135 and 155 bpm.

**Table 1:** Hemodynamic parameters in ascending aorta and femoral artery at baseline (80–105 bpm) and HR of 135 and 155 bpm.

**Animals**	**Ascending aorta**					
	**R (mmHg**·**s/ml)**			**C (ml/mmHg)**		
	**Baseline**	**135 bpm**	**155 bpm**	**Baseline**	**135 bpm**	**155 bpm**
1	1.83	1.77	1.88	0.54	0.25	0.27
2	1.37	1.38	1.37	0.48	0.40	0.39
3	0.87	1.19	1.16	0.97	0.54	0.52
4	0.48	0.46	0.43	1.41	0.98	1.05
5	0.56	0.75	0.84	0.59	0.27	0.18
6	1.62	1.64	1.65	0.44	0.37	0.36
Mean ± SD	1.21 ± 0.57	1.20 ± 0.51	1.22 ± 0.53	0.74 ± 0.38	0.47 ± 0.27	0.46 ± 0.31
*p*-value	0.26 (Baseline-135) 0.40 (135–155)			0.009 (Baseline-135) 0.77 (135–155)		
**Animals**	**Femoral artery**					
	**R (mmHg**·**s/ml)**			**C (ml/mmHg)**		
	**Baseline**	**135 bpm**	**155 bpm**	**Baseline**	**135 bpm**	**155 bpm**
1	41.32	40.63	46.37	0.023	0.014	0.011
2	27.68	25.67	31.82	0.032	0.022	0.025
3	29.72	30.01	34.09	0.043	0.025	0.028
4	24.23	24.91	29.32	0.033	0.026	0.025
5	21.31	22.30	22.51	0.029	0.018	0.018
6	41.50	41.69	42.24	0.021	0.020	0.017
Mean ± SD	30.96 ± 8.59	30.87 ± 8.36	34.39 ± 8.71	0.030 ± 0.008	0.021 ± 0.004	0.021 ± 0.006
*p*-value	0.85 (Baseline-135) 0.02 (135–155)			0.009 (Baseline-135) 0.89 (135–155)		

*R and C refer to the peripheral resistance and total arterial compliance, respectively*.

**Figure 3:**
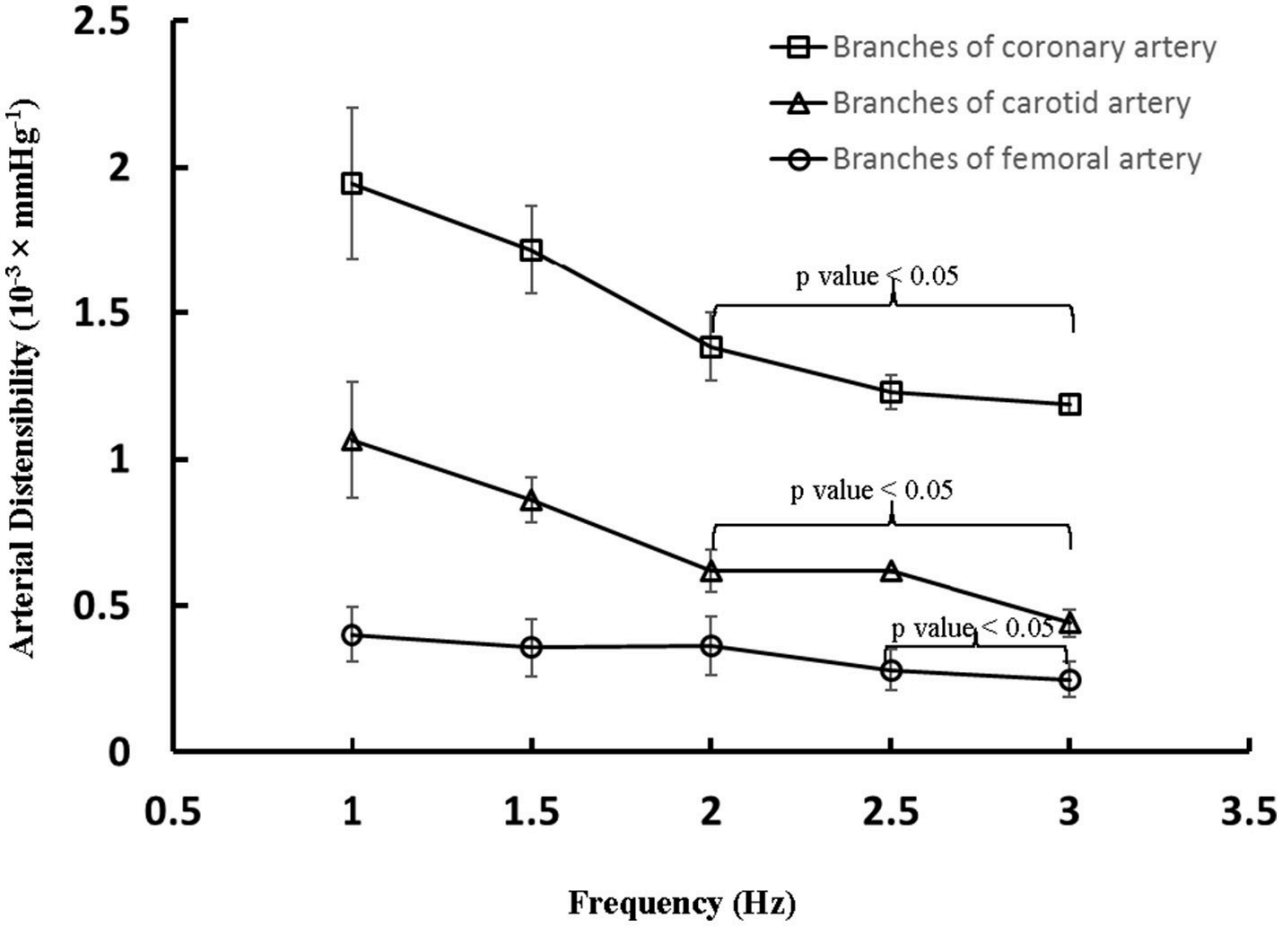
Arterial distensibility (Unit: 10^−3^ × mmHg^−1^) as a function of frequency in isolated small branches of coronary, carotid or femoral arteries. There is statistically significant difference (*p* < 0.05) in arterial distensibility between 1.5 Hz (mimicking the HR of 90 bpm) and higher frequencies in parentheses (i.e., ≥2 Hz in branches of coronary and carotid artery and ≥2.5 Hz in branches of femoral artery).

**Figure 4:**
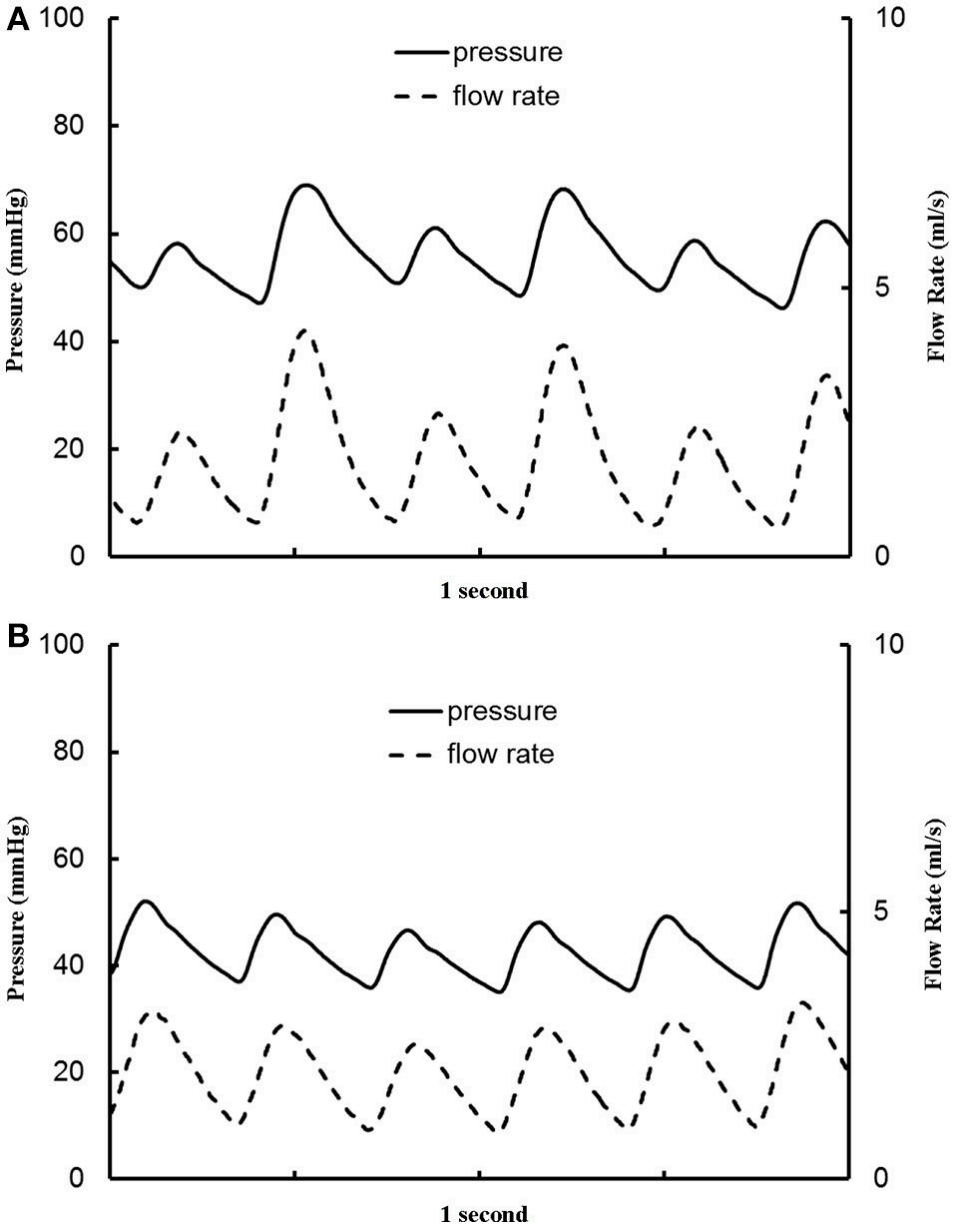
Pressure and flow waves in femoral artery **(A)** after the heart rate was paced to 170 bpm and **(B)** after the heart rate was paced to 170 bpm with I.V. injection of papaverine (100 mg per dose).

The aortic (carotid-femoral) PWV was reduced (*p* < 0.05) as HR increased from baseline to 135 bpm, as shown in Table 2. There was no statistical difference of PWV (*p* = 0.15) between 135 and 155 bpm. The characteristic impedance in the descending aorta had values of 0.31 ± 0.22, 0.27 ± 0.13, and 0.26 ± 0.13 mmHg·s/ml for baseline, 135, and 155 bpm, respectively. On the other hand, Figure 3 shows a decrease of arterial distensibility (Unit: 10^−3^ × mmHg^−1^) as the frequency increases in isolated small branches of coronary (square mark), carotid (triangle mark) or femoral (circle mark) arteries. There was statistically significant difference (*p* < 0.05) in arterial distensibility between 1.5 Hz (mimicking the HR of 90 bpm) and higher frequencies (i.e., ≥2 Hz in branches of coronary and carotid artery and ≥2.5 Hz in branches of femoral artery). Moreover, Figure 4A shows pressure and flow waves in femoral artery after HR was paced to 170 bpm. There are different amplitudes between consecutive heart beats. Figure 4B shows the corresponding waves in femoral artery after I.V. injection of papaverine (100 mg per dose). This restores successive amplitudes of pressure and flow waves.

**Table 2:** PWV along aorta from carotid to femoral arteries at baseline (80–105 bpm) and HR of 135 and 155 bpm.

**Animals**	**PWV along aorta from carotid to femoral arteries (m/s)**		
	**Baseline**	**135 bpm**	**155 bpm**
1	8.45	6.14	6.31
2	9.33	8.81	7.97
3	4.95	3.75	3.50
4	3.61	3.30	3.25
5	4.50	3.72	3.63
6	9.34	8.35	6.99
Mean ± SD	6.70 ± 2.62	5.68 ± 2.47	5.28 ± 2.06
*p*-value	0.017 (Baseline-135) 0.15 (135–155)		

*PWV refers to the pulse wave velocity*.

**Figure 5:**
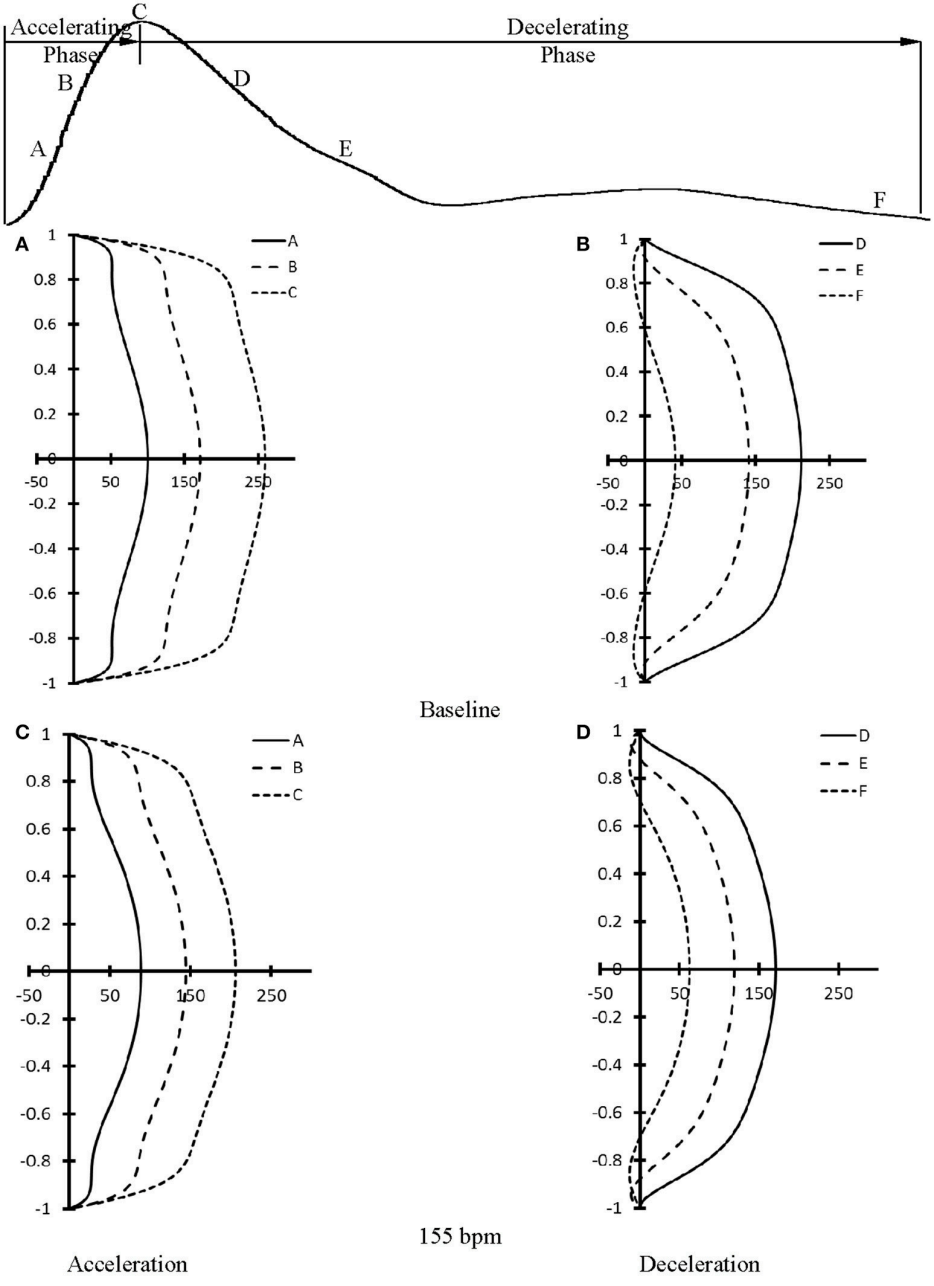
Flow velocity profiles in ascending aorta at various time instances during **(A)** accelerating and **(B)** decelerating periods at baseline. **(C,D)** refer to flow velocity profiles in ascending aorta after the heart rate was paced to 155 bpm in correspondence with **(A,B)**. (A–F) in **(A–D)** refer to time instances A–F as shown in the top curve.

**Figure 6:**
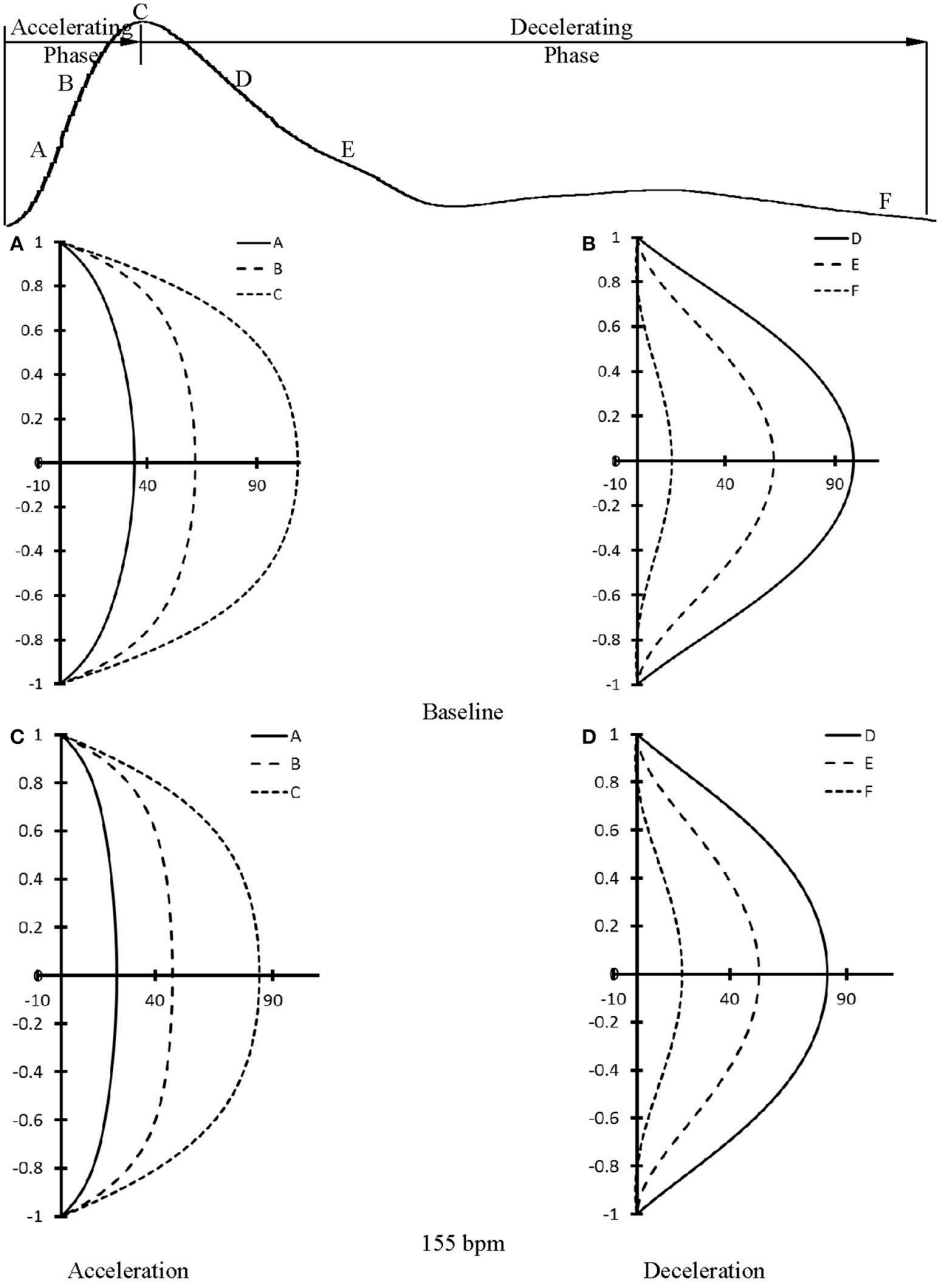
Flow velocity profiles in femoral artery at various time instances during **(A)** accelerating and **(B)** decelerating periods at baseline. **(C,D)** refer to flow velocity profiles in femoral artery after the heart rate was paced to 155 bpm in correspondence with **(A,B)**. (A–F) in **(A–D)** refer to time instances A–F as shown in the top curve.

At baseline, Figure 5A shows flow velocity profiles in ascending aorta along the accelerating period of flow waveform and Figure 5B shows flow velocity profiles along the decelerating period of flow waveform. Figures 5C,D show the corresponding flow velocity profiles at HR of 155 bpm. Peak Reynolds numbers (Re_peak_) are 6,000 and 4,400 for baseline and 155 bpm, respectively, while Womersley numbers (Wo) are 8.9 and 11.7. Moreover, Figures 6A–D show flow velocity profiles in femoral artery at baseline and 155 bpm. The femoral artery has peak Reynolds numbers of 690 and 530, and Womersley numbers of 3.2 and 4.2 for baseline and 155 bpm, respectively.

## Discussion

The major finding of the present study was that aortic PWV, determined by the validated techniques in Mohiaddin et al. (1993) and Butlin et al. (2013), and characteristic impedance decreased (i.e., the increase of aortic distensibility) as HR increased from baseline to 135 bpm. We also found that: (1) Total arterial compliance, SV, systemic PP, and central systolic pressure decreased by 36 ± 17, 38 ± 26, 29 ± 16, and 23 ± 12%, respectively, while the MAP, CO and peripheral resistance remained relatively unchanged as HR increased from baseline (HR of 80–105 bpm) to 135 bpm; (2) No statistical difference was found in hemodynamic parameters between 135 and 155 bpm; (3) Arterial distensibility decreased in small arteries as the frequency increased from 1.5 Hz (mimicking 90 bpm) to ≥2 Hz (mimicking HR ≥ 120 bpm); and (4) An increase of HR has relatively small effects on flow velocity profiles at HR range of 90–155 bpm, but results in an increase of the turnover of positive and negative WSS.

### Systemic hemodynamics

It has been documented that pharmacological HR lowering in patients with high BP can result in adverse cardiovascular outcomes (Rimoldi et al., 2016). Here, we showed significantly higher values of SV, systemic PP, and central systolic pressure at baseline as compared to HR of 135 and 155 bpm in swine. A high value of SV due to the prolonged LV (left ventricle) filling time increases LV preload and higher values of systemic PP and central systolic pressure increase LV afterload. This indicates that lowering HR should be considered cautiously in patients, which supports previous conclusion (Rimoldi et al., 2016).

The elevated HR by rapid atrial pacing from baseline to 135 bpm was found to significantly reduce the total arterial compliance (C in Equations 1, 2) distal to the ascending aorta, but remain the peripherical resistance (R in Equations 1, 2) in comparison with the baseline, as shown in Table 1 and Figure 1, which is consistent with a human study (Liang et al., 1999). A decrease of total arterial compliance has been shown to be chronically detrimental for the cardiovascular system (Westerhof et al., 2009). It is known that the total arterial compliance can be estimated by the equation C = (*SV*·*A*_*d*_)/[(*A*_*s*_+*A*_*d*_)(*P*_*ES*_−*P*_*d*_)], where *A*_*s*_ and *A*_*d*_ refer to the systolic and diastolic areas under the pressure curve (Liu et al., 1986). *P*_*d*_ refers to the diastolic pressure and *P*_*ES*_ refers to the end-systolic aortic pressure (or the pressure at the dicrotic notch). Since the value of *A*_*d*_/(*A*_*s*_+*A*_*d*_)/(*P*_*ES*_−*P*_*d*_) remains relatively unchanged, the 38 ± 26% decrease of SV is the major determinant for the 36 ± 17% decrease of total arterial compliance. In contrast, a decrease of systemic PP (29 ± 16%) due to the elevated HR may be beneficial for the cardiovascular system because a high value of systemic PP is known to correlate with cardiovascular mortality and morbidity (Benetos et al., 1997). In acute tachycardia, systemic PP is mainly determined by cardiac function while the total arterial compliance distal to the ascending aorta accounts for the entire systemic arteries (Nichols and McDonald, 2011). This suggests different mechanical responses of ventricle and vascular system in response to the rapid atrial pacing from baseline to 135 bpm, which requires further investigations. On the other hand, the relatively unchanged SV, systemic PP, and total arterial compliance as HR increases from 135 to 155 bpm imply a different equilibrium between the ventricle and vascular system from normal range of HR.

### Arterial distensibility

Acute tachycardia significantly affects the mechanical response of normal conduit arteries given the changes in systemic hemodynamics. We determined the aortic PWV using the foot-to-foot method suggested by expert consensus in European Heart Journal (Laurent et al., 2006). The aortic PWV was found to decrease (*p* < 0.05) with elevated HR from baseline to 135 bpm, which is consistent with the previous studies (Stefanadis et al., 1998; Wilkinson et al., 2000; Albaladejo et al., 2001). There was no statistical difference of PWV between 135 bpm and 155 bpm. The variation of characteristic impedance (Z_c_ in Equation 2) in descending aorta was consistent with the measured aortic PWV given Z_c_∞PWV (Huo and Kassab, 2006). Clinical studies have investigated the relationship between acute tachycardia and aortic PWV, which have led to contradictory results such as: unchanged PWV (Wilkinson et al., 2000; Albaladejo et al., 2001), decreased PWV (Stefanadis et al., 1998), or increased PWV (Liang et al., 1999; Lantelme et al., 2002; Haesler et al., 2004). Liang et al. found a significant increase of MAP and aortic PWV as patient's HR changed from 56 to 80 bpm, but no significant difference as HR increased from 80 to 100 bpm (Liang et al., 1999). Haesler et al. and Lantelme et al. showed constant MAP and increased PWV in elevated HR (the highest HR was below 100 bpm) in patients with a low degree of atherosclerosis (Haesler et al., 2004) and in subjects with a mean age of 77.8 ± 8.4 years (Lantelme et al., 2002), respectively. The increase of PWV by rapid pacing is not in agreement with the findings in Table 2 (normal swine), which may be attributed to aortic diseases such as atherosclerosis (Haesler et al., 2004) and old age (Lantelme et al., 2002) in those patients, or the role of activation of adrenergic system in physiological HR (from 56 to 80 bpm) in young patients not treated with sedative drugs (Liang et al., 1999). Moreover, MAP may be one of the most important factors to affect aortic PWV (Nichols and McDonald, 2011; Townsend et al., 2015). Some recent studies showed that HR dependency of PWV is different at high pressures than at low pressures and HR has a minimal influence on PWV in the lower range of MAP (Safar et al., 2003; Tan et al., 2012). Here, MAP is lower than 100 mmHg such that HR has relatively slight effect on PWV.

A significant decrease of the distensibility of coronary, carotid and femoral arteries (diameters of 300–600 μm) was found as the frequency increased from 1.5 to ≥ 2 Hz. The interaction of incident and reflected pressure and flow waves determines the actual waves, which depends on the mechanical properties of large elastic arteries, small muscular arteries, and small arterioles (Nichols and McDonald, 2011; Townsend et al., 2015). The peripheral resistance mainly resides in the arteriolar vessels in normal subjects (Chilian, 1991; Huo and Kassab, 2009). The acute tachycardia does not alter the vasoreactivity and mechanical response of the arteriolar bed given the relatively unchanged MAP and peripheral resistance in HR range of 90–155 bpm. In contrast, the irregular pressure and flow waves occurred after the heart was paced to 170 bpm, which disappeared after the injection of papaverine (a smooth muscle relaxant). We have shown that the increased vascular tone significantly increases the arterial stiffness in coronary arteries (Huo et al., 2012, 2013). These findings imply relatively small effects of vascular tone on the stiffness of small muscular arteries and arterioles in HR range of 90–155 bpm albeit the biology-regulated tone can affect vascular mechanical properties after the heart was paced to 170 bpm. Since the distensibility was determined in isolated small muscular arteries, *in vivo* measurements should be demonstrated to validate the implication. Here, we showed that pacing-induced acute tachycardia affects the elastic response of the arterial wall in small muscular arteries significantly as compared with the aorta, which is consistent with a previous study (Whelton et al., 2013). Moreover, the mismatch between aorta and small muscular arteries in high HR may be a risk factor for adverse cardiovascular events in the long term, which requires further investigations.

### Local hemodynamics

The accelerating and decelerating phases of pulsatile flow are clearly distinguishable periods in a cardiac cycle, as shown in top curves in Figures 5, 6. Since Re_peak_ (Re_peak_ > 4400) > 250·Wo (Wo < 11.7), the blood flow in aorta should result in turbulence during the decelerating phase of pulsatile flow, but not during the accelerating phase (Nerem and Seed, 1972). Since the present experimental techniques and analytic models did not assess turbulence, we used the Womersley model to simplify the flow velocity profiles. A near-wall flow reversal was found during the decelerating phase, but not during the accelerating phase. Moreover, the ascending aorta has a much larger value of peak Reynolds number than the femoral artery (6,000 vs. 690 at baseline), which leads to the less stable blunt flow velocity profiles as compared with the parabolic profiles in femoral artery. The increased HR, however, seems to have a negligible effect on the flow velocity profiles.

The low time-averaged WSS and high OSI are thought to result in endothelial dysfunction, monocyte deposition, smooth muscle cell proliferation, microemboli formation, and so on (Fan et al., 2016; Huang et al., 2016). In addition, the increased frequency of oscillatory shear stress can cause sustained molecular signaling of pro-inflammatory and proliferative pathways that contribute to endothelial dysfunction and atherosclerosis (Chien, 2008). Elevated HR increases the frequency of oscillatory shear stress while it has very small effects on the time-averaged WSS and OSI. This increased oscillatory shear may be a risk factor for the development of atherosclerosis in arteries, if sustained clinically.

### Critique of study

The arterial distensibility were measured in isolated small arteries with diameters of 300-600 μm, which needs to be incorporated into the cardiovascular system for a more systematic analysis. Hence, the hemodynamic analysis in the entire arterial tree including large arteries, small arteries and small arterioles should be implemented to help understand the contribution of both acute and chronic high HR to arterial stiffness and atherosclerosis (Huo and Kassab, 2006, 2007; Huo et al., 2009; Feng et al., 2018). In a review article (Safar et al., 2003), Safar et al. indicated that the aorta and its main branches are highly sensitive to age and changes in MAP while the small muscle arteries are highly sensitive to vasoactive substances (particularly those of endothelial origin). The cellular mechanisms of arterial stiffness in response to chronic high HR (particularly for the regulation of autonomic nervous system and endocrine system) need further investigations in relation to the remodeling of endothelial and smooth muscle cells.

## Conclusions

This study investigated the role of rapid pacing on systemic and local hemodynamics and arterial stiffness through *in vivo* and *ex vivo* experimental measurements and hemodynamic analysis. The acute increase in HR up to 135 bpm resulted in significant increase/decrease of the distensibility of aorta/small muscular arteries and a mismatch in the response of aorta and small arteries. The mismatch between aorta and small muscular arteries in high HR can be a risk factor for adverse cardiovascular events. The pacing also reduced the total arterial compliance, SV, systemic PP, and central systolic pressure but had no statistical significant effect on MAP, CO, peripheral resistance, and vascular flow patterns. Finally, pacing from 135 to 155 bpm had negligible effect on systemic hemodynamics and arterial stiffness.

## Author contributions

Data analysis were performed by YH at the college of engineering, Peking University and HC at the California Medical Innovations Institute. Paper was drafted and revised by YH and GK at the California Medical Innovations Institute.

### Conflict of interest statement

The authors declare that the research was conducted in the absence of any commercial or financial relationships that could be construed as a potential conflict of interest.
